# Supplementary material for: Reduced Toxicity of Trichothecenes, Isotrichodermol, and Deoxynivalenol, by Transgenic Expression of the Tri101 3-O-Acetyltransferase Gene in Cultured Mammalian FM3A Cells
Source: Toxins (Basel). 2019 Nov 10;11(11):654. doi: 10.3390/toxins11110654 (PMC6891669; doi:10.3390/toxins11110654)
Supplement: Supplementary file 1 [file toxins-11-00654-s001.pdf]

Supplementary Materials: Reduced Toxicity of Trichothecene by Transgenic Expression of *Tri101* 3-*O*-acetyltransferase Gene in Cultured Mammalian Cells

Nozomu Tanaka, Ryo Takushima, Akira Tanaka, Ayaki Okada, Kosuke Matsui, Kazuyuki Maeda, Shunichi Aikawa, Makoto Kimura and Naoko Takahashi-Ando

Table S1. The rate of deacetylation of 3-ADON and ITD after 48 h incubation under various conditions.

	H_2O	125 mM Tris-HCl buffer (pH 6.5)	RPMI1640 medium	N-medium (non-boiled FBS)	B-medium (boiled FBS)	FM3A cell culture in B-medium (1×10^5/ml)	FM3A cell culture in B-medium (6×10^5/ml)
3-ADON→DON	0.0%	1.7%	2.5%	4.6%	2.9%	4.9%	10.3%
ITD→ITDol	0.0%	2.1%	2.9%	96.4%	4.6%	17.0%	39.1%

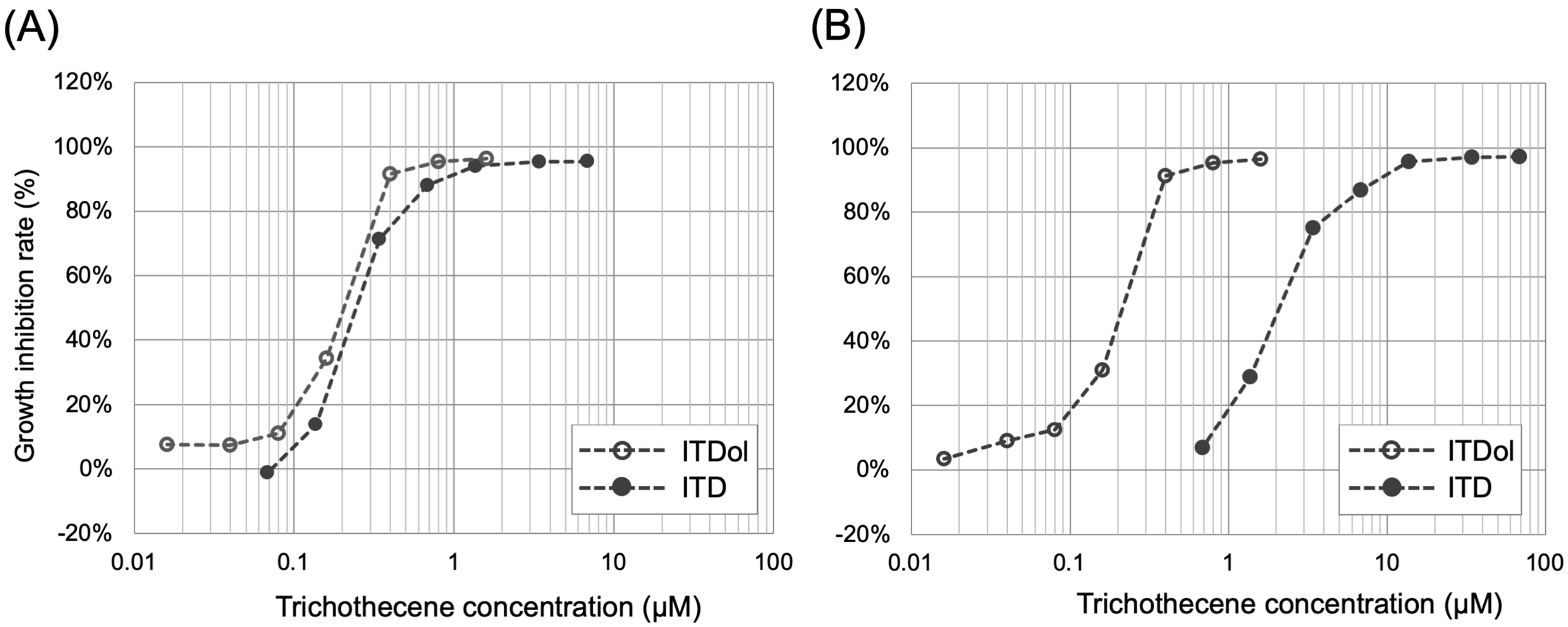

Figure S1. The dose-response cytotoxicity curves of trichothecenes. Cytotoxicity assay of ITDol and ITD on FM3A WT cells was carried out using CCK-8 reagent in 96-well plates. Cells were incubated (**A**) in N-medium and (**B**) in B-medium. Each trichothecene was prepared in 50% DMSO, and 1 μl of a toxin was added to 99 μl of cell culture. After 2-day incubation in a CO_2 incubator, 10 μl of CCK-8 reagent was added to each well and incubated at 37 ˚C for 3 h and OD_{450} of the solution in each well was measured using Multiskan™ FC plate reader. Growth inhibition (%) was calculated as 100 × {(OD_{450} of vehicle control – OD_{450} of background) - (OD_{450} of trichothecene added – OD_{450} of background)}/ (OD_{450} of vehicle control – OD_{450} of background).

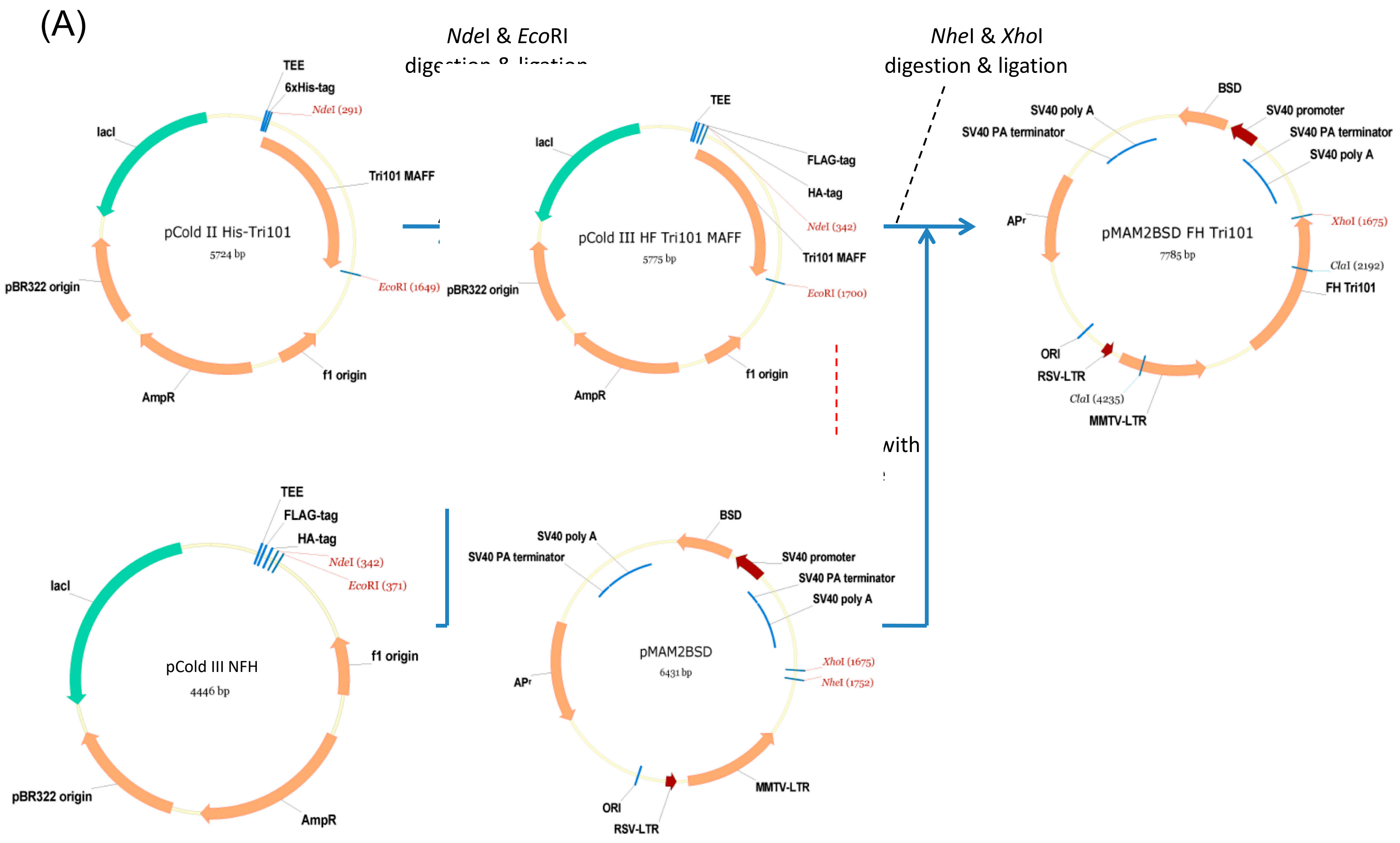
(A)
*Nde*I & *Eco*RI
*Nhe*I & *Xho*I
digestion & ligation
pCold II His-Tri101
5724 bp
TEE
6xHis-tag
NdeI (291)
lacI
Tri101 MAFF
EcoRI (1649)
pBR322 origin
AmpR
f1 origin
pCold III HF Tri101 MAFF
5775 bp
FLAG-tag
HA-tag
NdeI (342)
EcoRI (1700)
pMAM2BSD FH Tri101
7785 bp
BSD
SV40 poly A
SV40 PA terminator
SV40 promoter
APr
XhoI (1675)
ClaI (2192)
FH Tri101
ORI
RSV-LTR
ClaI (4235)
MMTV-LTR
with
pCold III NFH
4446 bp
NdeI (342)
EcoRI (371)
pMAM2BSD
6431 bp
NheI (1752)

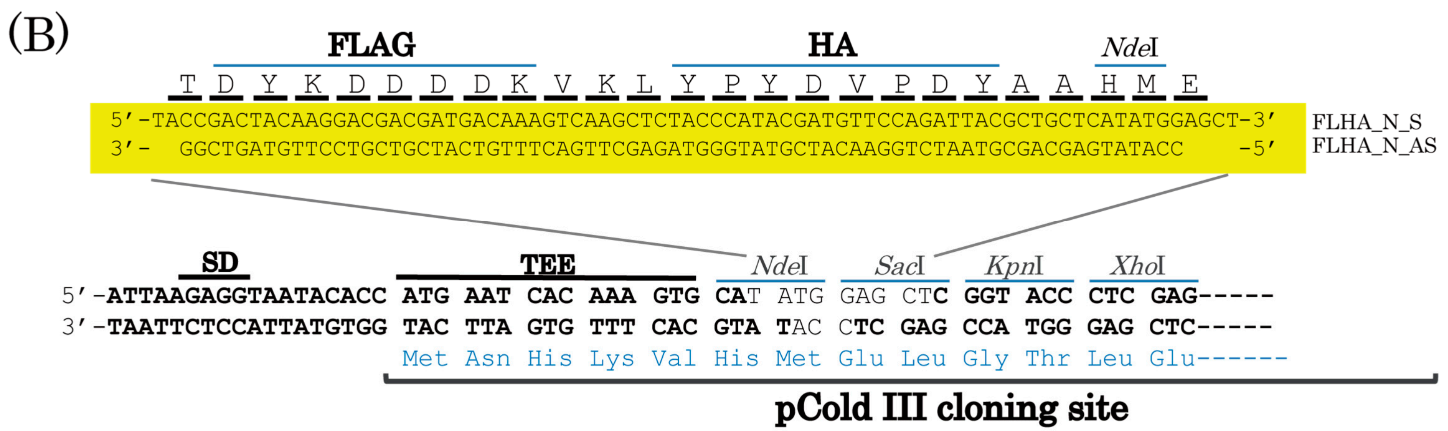

Figure S2. Construction of pMAM2BSD_FH_Tri101. (**A**) The plasmid pMAMBSD_FH_Tri101 was constructed as described in Materials and Methods. (**B**) The plasmid pColdIII-NFH was constructed via the insertion of synthetic oligonucleotides (highlighted in yellow) between *Nde*I and *Sac*I sites of pCold™III vector (Takara Bio Inc. Shiga, Japan).
